# Resveratrol protects podocytes against apoptosis via stimulation of autophagy in a mouse model of diabetic nephropathy

**DOI:** 10.1038/srep45692

**Published:** 2017-04-04

**Authors:** Shan-Shan Huang, Da-Fa Ding, Sheng Chen, Cheng-Long Dong, Xiao-Long Ye, Yang-Gang Yuan, Ya-Min Feng, Na You, Jia-Rong Xu, Heng Miao, Qiang You, Xiang Lu, Yi-Bing Lu

**Affiliations:** 1 Department of Endocrinology, The Second Affiliated Hospital of Nanjing Medical University, Nanjing Medical University, Nanjing, China; 2 Department of Endocrinology, Nanjing General Hospital of Nanjing Military Command, Nanjing, China; 3 Department of Emergency, Yancheng First People’s Hospital, Yancheng, China; 4 Department of Nephrology, The First Affiliated Hospital of Nanjing Medical University, Nanjing Medical University, Nanjing, China; 5 Department of Geriatics, The Second Affiliated Hospital of Nanjing Medical University, Nanjing Medical University, Nanjing, China

## Abstract

Podocyte apoptosis coincides with albuminuria onset and precedes podocytopenia in diabetic nephropathy. However, there is a lack of effective therapeutic drugs to protect podocytes from apoptosis. Here, we demonstrated that resveratrol relieved a series of indicators of diabetic nephropathy and attenuated apoptosis of podocytes in db/db diabetic model mice. In addition, resveratrol induced autophagy in both db/db mice and human podocytes. Furthermore, inhibition of autophagy by 3-methyladenine (3-MA) and autophagy gene 5 (Atg5) short hairpin RNA (shRNA) reversed the protective effects of resveratrol on podocytes. Finally, we found that resveratrol might regulate autophagy and apoptosis in db/db mice and podocytes through the suppression of microRNA-383-5p (miR-383-5p). Together, our results indicate that resveratrol effectively attenuates high glucose-induced apoptosis via the activation of autophagy in db/db mice and podocytes, which involves miR-383-5p. Thus, this study reveals a new possible strategy to treat diabetic nephropathy.

Diabetic nephropathy (DN) is a common chronic complication of diabetes characterized by increased urinary albumin excretion (microalbuminuria) and is currently the second leading cause of end-stage renal disease^1^. The early pathological changes of DN mainly include podocytes injury, detachment, and apoptosis, while surviving podocytes show compensatory hypertrophy and foot process fusion^2^.

Podocytes, which are visceral epithelial cells of the renal capsule, are attached to the outside of the glomerular basement membrane. This membrane, together with podocytes and the capillary endothelium, forms the glomerular filtration barrier. Podocytes are a type of terminally differentiated cells^3^. Multiple studies have proved that podocyte apoptosis coincides with albuminuria onset and precedes podocytopenia in different mouse models of diabetes^4^,^5^. At present, the treatment options for patients with clinical DN are very limited, and mainly include strict control of blood glucose, low-protein diet, the use of angiotensin II type 1 (AT1) receptor antagonists, angiotensin II-converting enzyme inhibitors, and other drugs^6^. However, there is a lack of effective therapeutic drugs to protect the cells from apoptosis.

Resveratrol (3′, 5′, 4′-trihydroxystibene) is a non-flavonoid polyphenol with various pharmacological effects, such as free-radical scavenging, anti-inflammatory, and antitumor effects^7^,^8^. It has attracted increased research attention in the field of DN for its potential value in kidney protection. Our previous studies have suggested that resveratrol exerts antiproliferative and antihypertrophic effects by activating adenosine 5′-monophosphate (AMP)-activated protein kinase (AMPK) and reducing 4E-BP1 and S6 phosphorylation, thus suppressing the development and progression of DN^9^. Chuan-Ming Hao *et al*.^10^ demonstrated that resveratrol could attenuate DN via inhibiting the VEGF-Flk-1 system. They found treatment with resveratrol not only suppressed the expression levels of VEGF and Flk-1 in the diabetic rat kidneys, but also inhibited VEGF expression and secretion in cultured podocytes. Besides, Aihua Zhang *et al*.^11^ found resveratrol attenuated aldosterone-induced mitochondrial malfunction and podocyte injury *in vitro* and in aldosterone-infused mice *in vivo*.

Autophagy is a lysosome-dependent protein degradation pathway that functions in both physiological and pathological processes of the body. During the autophagic process, cells form an autophagic body through the inclusion of degradation products. The autophagic body is transported to the lysosome for digestion and degradation by a variety of enzymes to recycle degradation products and maintain intracellular homeostasis^12^. More and more studies demonstrated the protective role of podocyte autophagy *in vivo*^13^. Tharaux *et al*.^14^ found endothelial cell and podocyte autophagy synergistically protect from diabetes-induced glomerulosclerosis in a model of type I diabetic nephropathy. Besides, Uzu T *et al*.^15^ demonstrated that impaired podocyte autophagy exacerbates proteinuria in type II diabetic nephropathy models. Similarly, our previous studies also revealed that high glucose (HG) leads to increased autophagy in podocytes at an early stage and that autophagy is a prosurvival mechanism under these conditions^16^.

MicroRNAs (MiRNAs) are ~22 nucleotide-long noncoding RNAs and recognize specific sequences termed miRNA-response elements that are generally present in the 3′-untranslated region (3′ UTR) of target mRNAs. MiRNAs play an important role in the process of cell differentiation, biological development and disease development^17^, interestingly, miRNAs have been shown to play a central role in autophagy regulation^18^,^19^.

Glomerular podocytes are highly specialized epithelial cells whose injury in glomerular diseases causes proteinuria, so it is important to protect them as early as possible. Although there are some studies on the protective effect of resveratrol on podocytes, however, until date, no study on resveratrol-mediated autophagy in podocytes has been conducted and the mechanism of autophagy in podocytes has not been completely revealed. Here, we first investigated the effects of resveratrol on the progression of DN and the apoptosis of podocytes in db/db mice and cultured human podocytes. Then, we explored whether resveratrol improved the level of autophagy in the db/db mice and podocytes. In particular, to determine the effect of autophagy on HG-mediated apoptosis in resveratrol-treated podocytes, we inhibited autophagy by using the inhibitor 3-MA and by transfecting cells with Atg5 shRNA. Finally, we identified miRNAs differentially expressed in normal and DN tissues by microarray analysis of mouse kidney samples to investigate whether resveratrol regulates autophagy and apoptosis in db/db mice and human podocytes through the suppression of miRNA.

## Results

### Resveratrol relieves a series of indicators of DN and attenuates apoptosis of podocytes in db/db mice

**Figure 1:**
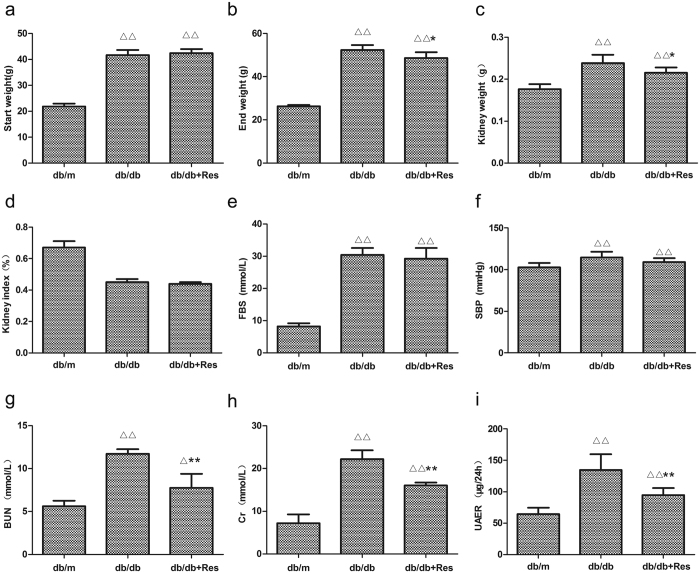
Resveratrol relieves a series of indicators of DN. (**a**–**i**) Quantitative analysis of body weight, kidney weight, kidney index, FBS, SBP, BUN, Cr, and UAER in three groups. Data are the mean ± SEM of three experiments. ∆*P* < 0.05 vs. db/m, ∆∆*P* < 0.01 vs. db/m, *P < 0.05 vs. db/db, **P < 0.05 vs. db/db.

To investigate the potential therapeutic effect of resveratrol on DN in a db/db diabetic mouse model and to determine whether resveratrol attenuates apoptosis in the db/db mouse kidney, three groups of mice were used: control db/m mice, db/db mice, and resveratrol-treated db/db mice (db/db + Res) (n = 6 per group). Figure 1 list detailed characteristics of the three groups. The body and kidney weights were significantly higher in db/db mice than in db/m mice throughout the experiment. In db/db mice, treatment with resveratrol tended to lead to lower body and kidney weights by the end of the experiment (Fig. 1a–c). However, there was no difference in the kidney index (body weight/kidney weight) between untreated and resveratrol-treated db/db mice (Fig. 1d). Additionally, the fasting blood glucose (FBS) was higher in db/db than in db/m mice; however, no significant difference was noted in the level of fasting blood glucose (FBS) of db/db mice with and without resveratrol treatment (Fig. 1e). The Systolic blood pressure (SBP) was higher in db/db mice than in db/m mice, however, SBP was not significantly different between db/db mice and resveratrol-treated db/db mice (Fig. 1f). In addition, 24-h microalbuminuria (UAER), serum creatinine (Cr), and urea nitrogen (BUN) were all higher in the db/db than in the control mice, while these parameters were significantly decreased by treatment with resveratrol, indicating that resveratrol ameliorates the DN-associated functional abnormalities in db/db mice (Fig. 1g–i).

**Figure 2:**
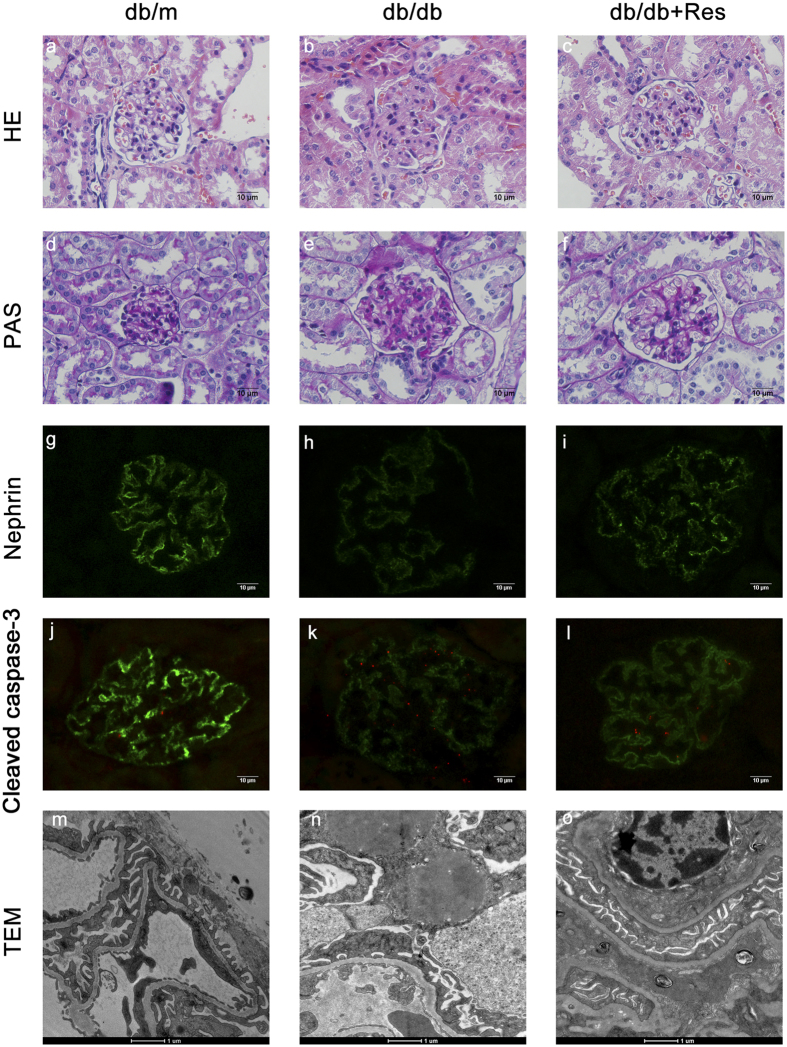
Treatment with resveratrol improves glomerular histological abnormalities in db/db mice. (**a**–**c**) Representative photomicrographs of HE-stained kidney sections. Magnification: 400×. (**d**–**f**) Representative photomicrographs of PAS-stained kidney sections. Magnification: 400×. (**g**–**l**) Representative photomicrographs of immunofluorescence for nephrin. Magnification: 400×. (**j**–**l**) Representative photomicrographs of immunofluorescence for Cleaved caspase-3. Cleaved caspase-3 exhibits red fluorescence in the renal glomeruli. Original magnification: 400×. (**m**–**o**) Electron microscopy of kidney sections. Magnification: 20,000×; scale bar: 1 μm. Data are results of experiments in each group (n = 6).

To determine the role of resveratrol in relieving db/db mice from DN further, kidney tissues were stained with hematoxylin-eosin (HE) and periodic acid-Schiff (PAS), and the expression of nephrin and Cleaved caspase-3 in the kidney was detected by immunohistochemistry and immunofluorescence. Figure 2a–f showed representative photomicrographs of renal glomeruli in PAS- and HE-stained kidney sections of the three groups. Renal tissues of db/db mice (Fig. 2b and e) showed a series of indicators of DN, such as glomerulus hypertrophy, capillary basement membrane thickening, and mesangial matrix expansion as compared to db/m mice (Fig. 2a and d). After treatment with resveratrol, glomerular lesion formation was remarkably alleviated (Fig. 2c and f). Nephrin also showed lower expression in renal glomerular expression in db/db than in db/m mice as indicated by immunofluorescence (Fig. 2g–i), while treatment with resveratrol restored expression in db/db mice. Cleaved caspase-3, a marker of apoptosis, is located in both the cytoplasm and the nuclei of the renal glomeruli. As shown in Fig. 2j and k, the expression level of cleaved caspase-3 was significantly higher in db/db than in db/m mice, while treatment with resveratrol reversed the higher expression in db/db mice (Fig. 2k and l). Transmission electron microscopy revealed fusion of the foot process and glomerular basement membrane in db/db mice as compared with db/m mice, while the damage of podocytes was alleviated after treatment with resveratrol (Fig. 2m–o). These results suggest that resveratrol improves histological abnormalities including glomerulus hypertrophy, capillary basement membrane thickening, and mesangial matrix expansion, and reduces DN-induced apoptosis in renal glomeruli. Moreover, resveratrol may play a role in the protection of podocytes.

### Resveratrol attenuates HG-induced apoptosis in human podocytes

**Figure 3:**
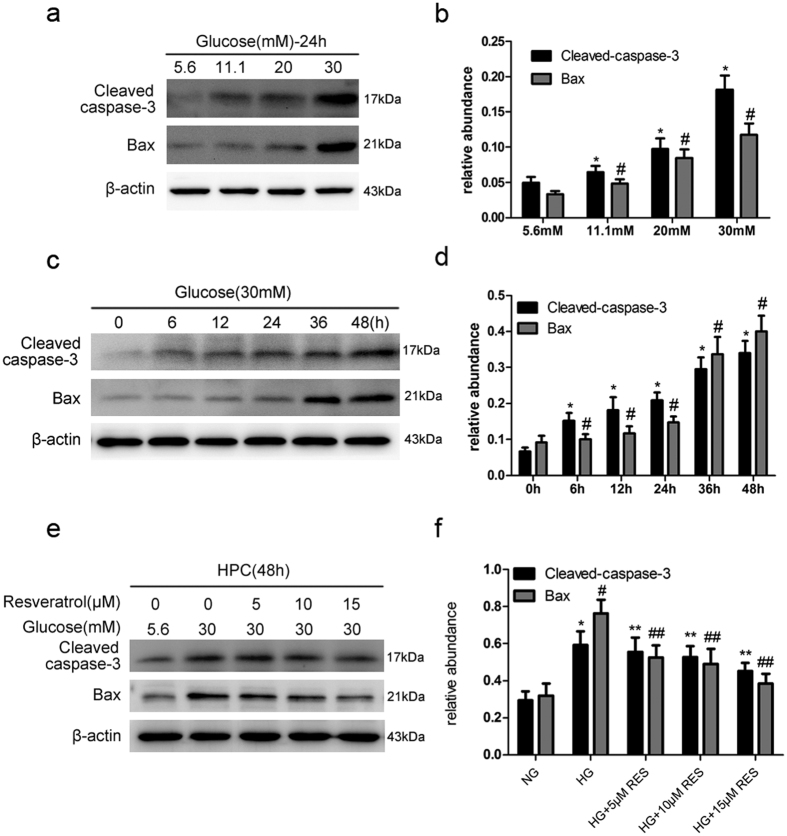
Resveratrol attenuates HG-induced apoptosis. Cell lysates were immunoblotted with antibodies against Cleaved caspase-3, Bax and β-actin. (**a**) Cells were treated with the indicated concentrations of glucose (5.6, 11.1, 20, and 30 mM) for 24 h. (**b**) Relative abundances of Cleaved caspase-3 and Bax normalized against β-actin. Data are the mean ± SEM of three experiments. *P < 0.05 vs. 5.6 mM glucose (NG), ^#^*P* < 0.05 vs. NG. (**c**) Cells were treated with HG (30 mM) for the indicated time points (0, 6, 12, 24, 36, and 48 h). (**d**) Relative abundances of Cleaved caspase-3 and Bax normalized to that of β-actin. Data are the mean ± SEM of three experiments. *P < 0.05 vs. control (0 h), ^#^*P* < 0.05 vs. control (0 h). (**e**) Human podocytes (HPC) were treated with various concentrations of resveratrol (0, 5, 10, and 15 μM) followed by HG treatment for 48 h. (**f**) Quantitative densitometry for Cleaved caspase-3 and Bax. Protein levels were normalized to that of β-actin. Data are the mean ± SEM of three experiments. *P < 0.05 vs. NG, ^#^*P* < 0.05 vs. NG, **P < 0.05 vs. HG, ^##^*P* < 0.05 vs. HG.

Because we observed that resveratrol could relieve various DN indicators and attenuate podocyte apoptosis—which may perform a specific function in podocytes of db/db mice, next, we set out to determine whether resveratrol attenuates HG-mediated apoptosis in human podocytes (HPC). Human podocytes were incubated with HG (5.6–30 mmol/l) for 24 h. Apoptosis induction was determined by western blotting. Cleaved caspase-3 and Bax, specific apoptosis makers, were upregulated in a dose-dependent manner and peaked at 30 mmol/l (Fig. 3a). Based on these results, we selected 30 mM as a stimulatory concentration for a time-course analysis of protein expression. Expression of both markers significantly increased in a time-dependent manner (Fig. 3c). These data suggest that HG induces podocyte apoptosis in a dose- and time-dependent manner. To investigate whether resveratrol attenuates HG-induced apoptosis in the human podocytes (HPC), the cells were treated with different concentrations of resveratrol (0, 5, 10, and 15 μM) followed by HG (30 mM) treatment for 48 h. The expression of Cleaved caspase-3 and Bax were assessed by western blotting. As shown in Fig. 3e, treatment with resveratrol strongly decreased expression of Cleaved caspase-3 and Bax in a dose-dependent manner.

**Figure 4:**
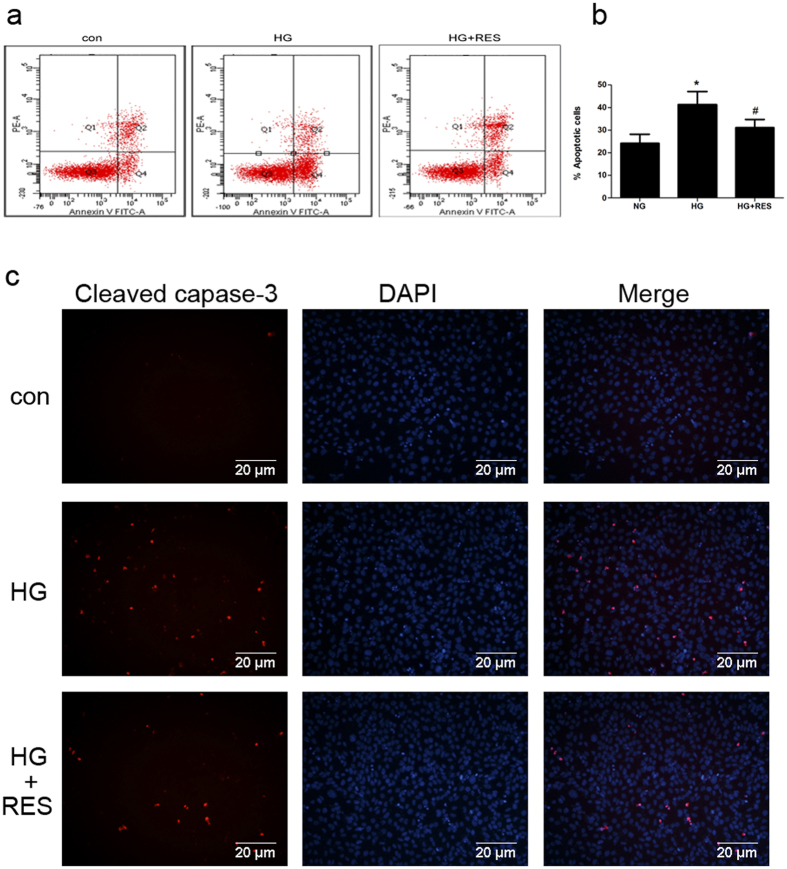
Resveratrol attenuates HG-induced apoptosis. (**a**) Cells were incubated in medium containing NG (5.6 mM), HG (30 mM), and HG in the presence of resveratrol (15 μM) for 48 h. The effects of resveratrol on HG-induced apoptosis were determined by flow cytometry. (**b**) Quantitative analysis of Annexin V+ PI− and Annexin V+ PI+ podocytes. Data are the mean ± SEM of three experiments. *P < 0.05 vs. NG, ^#^*P* < 0.05 vs. HG. (**c**) Representative images of immunofluorescence staining indicating the activation and cleavage of caspase-3. Cleaved caspase-3 exhibits red fluorescence and nuclei are stained with 4′,6-diamidino-2-phenylindole (DAPI; blue). Podocytes were treated with NG, HG, or HG plus resveratrol for 48 h. Magnification: 200×.

The effect of resveratrol on HG-induced apoptosis was further evaluated by flow-cytometric analysis of Annexin V-FITC- and propidium iodide (PI)-dual-stained cells. In Fig. 4a, Q1 indicated necrotic cells (Annexin−/PI+), Q2 indicated late apoptotic cells (Annexin+/PI+), Q3 indicated normal cells (Annexin−/PI−), and Q4 indicated early apoptotic cells (Annexin+/PI−). Podocytes were treated with normal glucose, HG, and HG plus resveratrol for 48 h. The number of apoptotic cells was increased by HG, and normalized by resveratrol pretreatment (Fig. 4a and b). Immunofluorescence microscopy confirmed that the expression of Cleaved caspase-3 was markedly elevated after HG stimulation, while pretreatment with resveratrol inhibited the effect (Fig. 4c). Collectively, these results indicate that resveratrol attenuates HG-induced apoptosis in human podocytes.

### Resveratrol induces autophagy in both db/db mice and human podocytes

**Figure 5:**
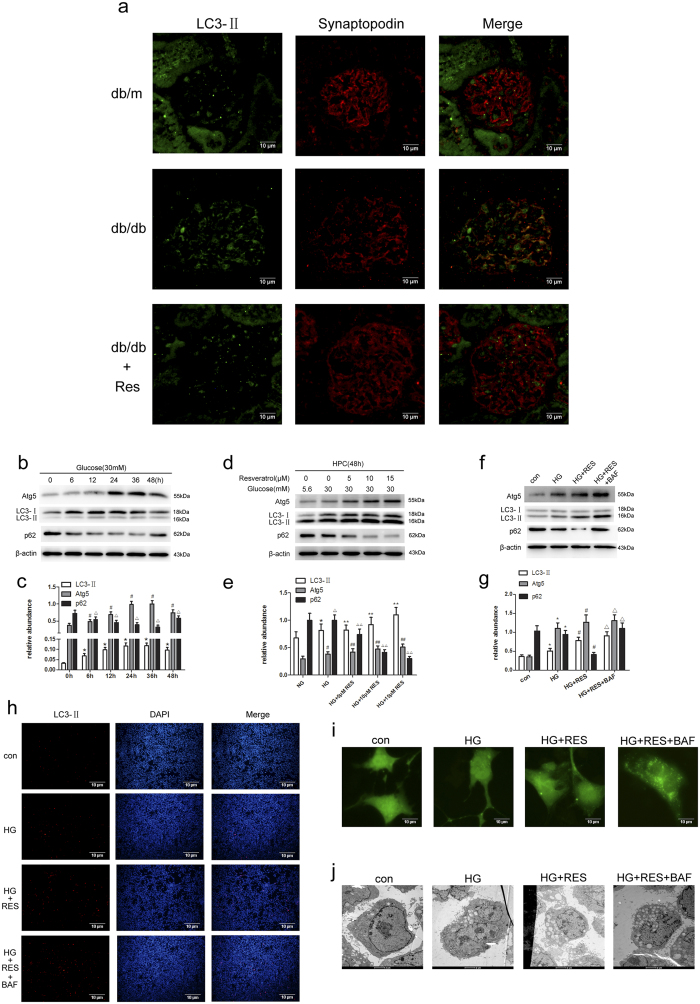
Resveratrol-induced autophagy in db/db mice and podocytes. (**a**) Representative photomicrographs of immunofluorescence for LC3-II and synaptopodin in mouse renal glomeruli. LC3-II exhibits green fluorescence and synaptopodin exhibits red fluorescence. Magnification: 400×. (**b–g**) Immunoblot and densitometric analysis of LC3, Atg5, p62 and β-actin in cells treated with indicated treatments for 48 h. (**b**) Cells were treated with HG (30 mM) for the indicated time points (0, 6, 12, 24, 36, and 48 h). (**c**) Quantitative densitometry for LC3-II, Atg5, and p62. Protein levels were normalized to that of β-actin. Data are the mean ± SEM of three experiments. *P < 0.05 vs. control (0 h), ^#^*P* < 0.05 vs. control (0 h), △*P* < 0.05 vs. control (0 h). (**d**) Human podocytes (HPC) were treated with different concentrations of resveratrol (0, 5, 10, and 15 μM) followed by HG treatment for 48 h. (**e**) Relative abundances of LC3-II, Atg5, and p62 normalized to that of β-actin. Data are the mean ± SEM of three experiments. *P < 0.05 vs. NG, ^#^*P* < 0.05 vs. NG, △*P* < 0.05 vs. NG, **P < 0.05 vs. HG, ^##^*P* < 0.05 vs. HG, △△*P* < 0.05 vs. HG. (**f**) Human podocytes (HPC) were treated with NG (5.6 mM), HG, HG plus resveratrol (15 μM), HG plus resveratrol (15 μM) and bafilomycin A (10 nM) for 48 h. (**g**) Relative abundances of LC3-II, Atg5, and p62 normalized to that of β-actin. Data are the mean ± SEM of three experiments. *P < 0.05 vs. NG, ^#^*P* < 0.05 vs. HG, △*P* < 0.05 vs. HG + RES. (**h**) Representative immunofluorescence staining for LC3-II. Podocytes were treated with NG (5.6 mM), HG, HG plus resveratrol (15 μM), HG plus resveratrol (15 μM) and bafilomycin A (10 nM) for 48 h. Magnification: 100×. (**j**) Electron microscopic evaluation of autophagy in podocytes. Equal numbers of human podocytes were incubated in medium containing NG, HG, HG plus resveratrol, HG plus resveratrol and bafilomycin A for the indicated periods. Magnification: 20,000×; scale bar, 1 μm. The numbers of autophagosomes represent autophagic activity. (**i**) Representative microphotographs of GFP-LC3-transfected podocytes incubated in medium containing NG, HG, HG plus resveratrol, HG plus resveratrol and bafilomycin A for the indicated periods. Magnification: 400×.

To examine the role of autophagy in db/db mice after treatment with resveratrol, immunofluorescence assay was performed to analyze the expression of LC3-II, the autophagic form of LC3. The expression of LC3-II and synaptopodin in db/db mice were lower than that in db/m mice, while treatment with resveratrol reversed the lower expression (Fig. 5a). This suggest that resveratrol ameliorates the abnormal autophagy level in db/db mice.

To observe how the level of autophagy changes with time by HG treatment, human podocytes were incubated with 30 mM glucose for various periods (0, 6, 12, 24, 36, and 48 h). Western blotting was used to detect the expression levels of autophagy-related proteins (LC3-II, Atg5 and p62). LC3-II and Atg5 were up-regulated as early as 6 h, and both declined dramatically at 48 h, on the contrary, p62 was increased significantly at 48 h (Fig. 5b). These results demonstrated that autophagy was initially up-regulated in podocytes, while it declines with treatment time.

To determine whether autophagy was stimulated in podocytes after exposure to resveratrol, four different approaches were employed: western blotting for autophagy-related proteins, detection of LC3-II by immunofluorescence staining, transmission electron microscopy, and evaluation of green fluorescent protein (GFP)-microtubule-associated protein 1 light chain 3 (LC3) punctate structures by fluorescence microscopy. LC3-II is present on isolation membranes and autophagosomes. Therefore, an increase in the level of cellular LC3-II reflects activation of autophagy. Cells were treated with different concentrations of resveratrol (0, 5, 10, and 15 μM), followed by HG (30 mM) treatment for 48 h. As shown in Fig. 5d, resveratrol induced a dose-dependent accumulation of LC3-II and Atg5 in podocytes as indicated by western blotting, on the contrary, treatment with resveratrol strongly decreased expression of p62. Next, cells were divided into four groups: normal glucose (NG: 5.6 mM), HG (30 mM), HG plus resveratrol (15 μM), HG plus resveratrol (15 μM) and bafilomycin A (10 nM). As shown in Fig. 5h, autophagosomes (stained with the anti-LC3-II antibody, red) were localized in the cytoplasm, and in cells treated with HG plus resveratrol and bafilomycin A (10 nM), the number of red fluorescent spots were highest among all the groups. Transmission electron microscopy is the gold standard for detecting autophagy; the number of autophagosomes represents autophagic activity. HG could induce autophagy and resveratrol further increased the number of autophagosomes (Fig. 5j). Finally, the widely used fusion protein GFP-LC3 was employed to monitor autophagy. Podocytes were transfected with GFP-LC3 plasmid and treated as mentioned above. As shown in Fig. 5i, GFP-LC3 green dots showed the highest accumulation after exposure to bafilomycin A although resveratrol also increased the number of green dots. Conversely, the fluorescence was predominantly diffuse in the cytoplasm of control cells.

### Inhibition of autophagy by 3-MA and Atg5 shRNA reverses the protective effects of resveratrol on human podocytes

**Figure 6:**
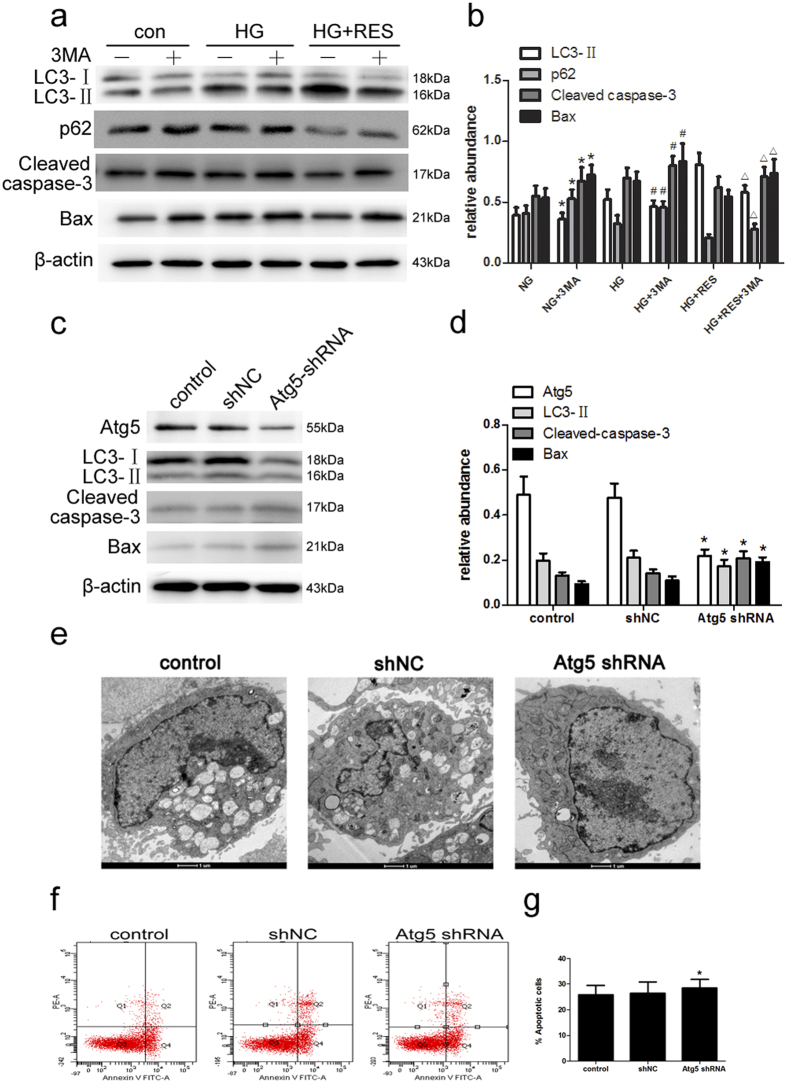
Inhibition of autophagy by 3-MA and increased apoptosis by Atg5 shRNA during incubation of podocytes with HG plus resveratrol. (**a**) Cells were pretreated with the indicated concentrations of 3-MA for 1 h before treatments for 48 h. Immunoblot analysis of LC3, p62, Cleaved caspase-3, and Bax in cells treated with the indicated treatments for 48 h. (**b**) Quantitative densitometry for LC3-II, p62, Cleaved caspase-3, and Bax. Data are the mean ± SEM of three experiments. *P < 0.05 vs. NG; ^#^*P* < 0.05 vs. HG; ∆*P* < 0.05 vs. HG + RES. (c-g) Podocytes were transfected with Atg5 shRNA or scrambled shRNA (control; shNC); 24 h after transfection, the cells were treated with HG plus resveratrol for 48 h. (**c**) Cell lysates were prepared and subjected to immunoblot analysis with antibodies for Atg5, LC3, Cleaved caspase-3, and Bax, with β-actin serving as a loading control. (**d**) Quantitative densitometry for Atg5, LC3-II, Cleaved caspase-3, and Bax. Protein levels were normalized to that of β-actin. Data are the mean ± SEM of three experiments. *P < 0.05 vs. shNC. (**e**) Electron microscopic evaluation of autophagy in podocytes in different treatment groups. (**f**) The effects of Atg5 shRNA on HG-induced apoptosis with resveratrol treatment were determined by flow cytometry. (**g**) Quantitative analysis of Annexin V+ PI− and Annexin V+ PI+ podocytes by flow cytometry. Data are the mean ± SEM of three experiments. *P < 0.05 vs. shNC.

To examine the role of autophagy in podocytes after exposure to HG and resveratrol, autophagy was inhibited chemically with 3-MA and with Atg5 shRNA. Cells were pretreated with 3-MA for 2 h followed by HG plus resveratrol for 48 h. As expected, 3-MA attenuated LC3-II expression and led to increases in p62, Cleaved caspase-3 and Bax expressionin the presence or absence of resveratrol (Fig. 6a). To further confirm these results, cells were transfected with Atg5 shRNA and subjected to the same treatment. Transfection with Atg5 shRNA reduced Atg5 and LC3-II expression as indicated by immunoblot analysis (Fig. 6c). In addition, Atg5 inhibition enhanced caspase-3 cleavage and Bax expression (Fig. 6c). Similarly, flow cytometric analysis showed that Atg5 inhibition significantly increased apoptotic cells in the case of resveratrol treatment (Fig. 6e). Meanwhile, immunofluorescence showed that suppression of Atg5 enhanced Cleaved caspase-3 expression (Fig. 6f). Taken together, these findings confirmed that resveratrol attenuates HG-induced apoptosis in podocytes via the activation of autophagy.

### Resveratrol regulates autophagy and apoptosis in db/db mice and human podocytes through the suppression of miR-383-5p

**Figure 7:**
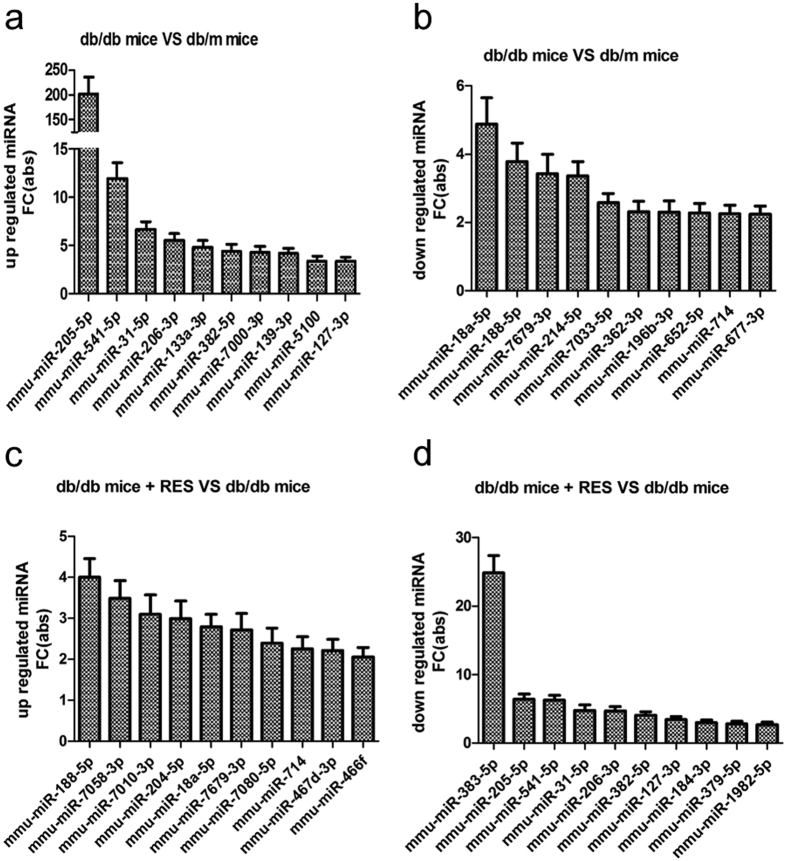
Differentially expressed miRNAs identified by microarray analysis. (**a**) MiRNAs up-regulated in db/db mice as compared with db/m mice. (**b**) MiRNAs down-regulated in db/db mice as compared with db/m mice. (**c**) MiRNAs up-regulated in resveratrol-treated db/db mice compared with db/db mice. (**d**) MiRNAs down-regulated in resveratrol-treated db/db mice compared with db/db mice.

**Figure 8:**
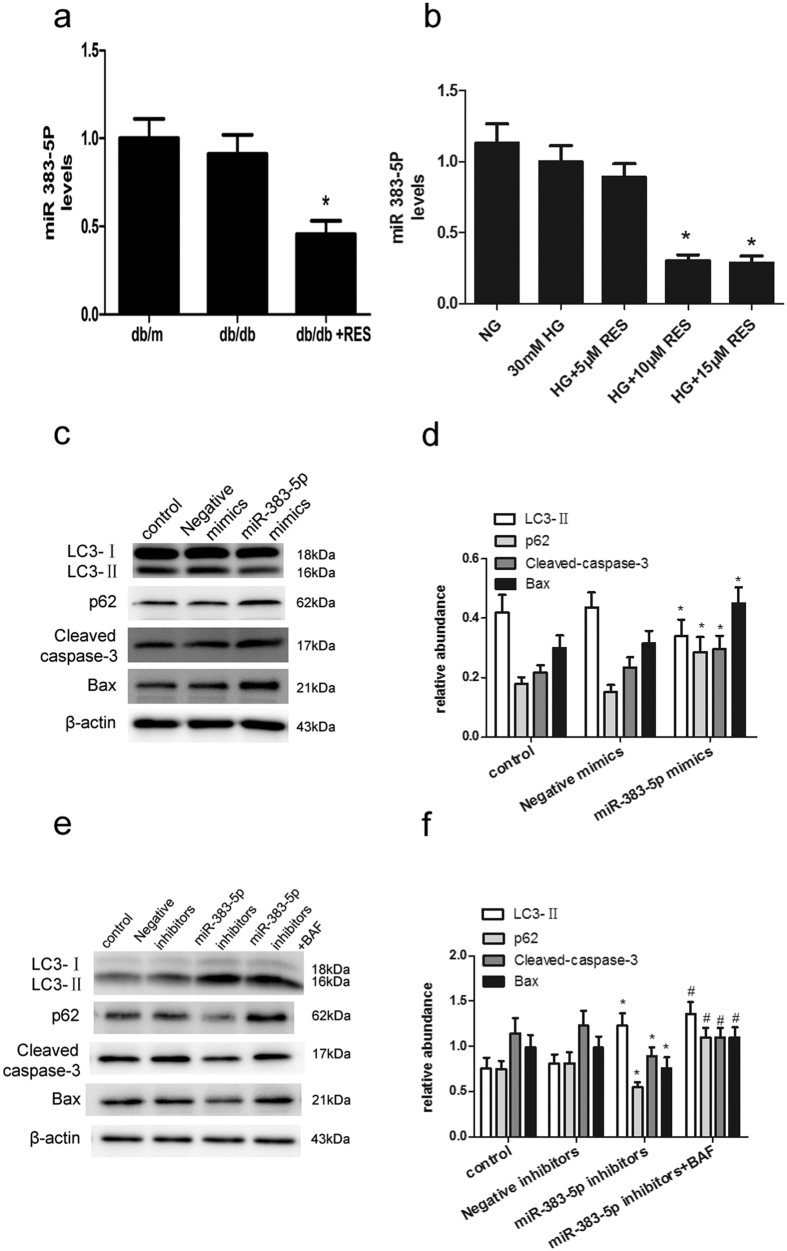
Resveratrol regulates autophagy and apoptosis in db/db mice and human podocytes through the suppression of miR-383-5p. (**a**) The expression of miR-383-5p in db/m, db/db, and resveratrol-treated db/db mice. Data are the mean ± SEM of three experiments. *P < 0.05 vs. db/db. (**b**) The expression of miR-383-5p at different concentrations of resveratrol (0, 5, 10, and 15 μM) and HG (30 mM), NG (5.6 mM) in podocytes. Data are the mean ± SEM of three experiments. *P < 0.05 vs. HG (30 mM). (**c**) Podocytes were transfected with miR-383-5p mimics or Negative mimics. Twenty-four hours after transfection, the cells were treated with HG plus resveratrol (15 μM) for 48 h. Cell lysates were subjected to immunoblot analysis with antibodies for LC3, p62, Cleaved caspase-3, and Bax, with β-actin serving as a loading control. (**d**) Quantitative densitometry for LC3-II, Cleaved caspase-3, and Bax. Relative protein levels were normalized to that of β-actin. Data are the mean ± SEM of three experiments. *P < 0.05 vs. Negative mimics. (**e**) Podocytes were transfected with miR-383-5p inhibitors or Negative inhibitors. Twenty-four hours after transfection, the cells were treated with HG plus resveratrol (15 μM), bafilomycin A for 48 h. Cell lysates were prepared and subjected to immunoblot analysis with antibodies for LC3, p62, Cleaved caspase-3, and Bax, with β-actin serving as a loading control. (**f**) Quantitative densitometry for LC3-II, p62, Cleaved caspase-3, and Bax. Relative protein levels were normalized to that of β-actin. Data are the mean ± SEM of three experiments. *P < 0.05 vs. Negative inhibitors. ^#^*P* < 0.05 vs. miR-383-5p inhibitors.

Mice kidney samples were subjected to microarray analysis to identify differentially expressed (fold-change ≥2) miRNAs. The results are shown in Fig. 7. From the differentially expressed miRNAs, we found when db/db mice treated with resveratrol (db/db + RES), the expression of miR-383-5p caused the most obvious decrease relative to db/db mice. So, we selected the most downregulated one, miR-383-5p to further identify its expression level in mice kidney samples and human podocytes under the indicated treatments. As shown in Fig. 8a, the expression of miR-383-5p was downregulated after treatment with resveratrol; however, there was no difference between db/m and db/db mice. In consistence, miR-383-5p was significantly decreased in podocytes after the addition of resveratrol with the dose of 10 μM and15 μM (Fig. 8b).

Next, we checked whether miR-383-5p had an effect on autophagy and apoptosis under HG plus resveratrol treatment. As shown in Fig. 8c, overexpression of miR-383-5p significantly blocked the increase in autophagy and attenuation of HG-induced apoptosis induced by resveratrol. While, treatment with bafilomycin A still increased the expression of p62 and LC3-II in podocytes although the suppression of miR-383-5p decreased the level of p62 (Fig. 8e).

## Discussion

Autophagy is considered a survival mechanism induced by various stimuli inadverse conditions to maintain cell integrity. Extensive studies have indicated that it plays important podocyte-protective roles in multiple kidney diseases, including DN. Compelling evidence has shown that resveratrol is capable of inducing autophagy in different cancer cell lines^20^,^21^,^22^; however, nothing is known about its effect on autophagy induction in renal cells. In this study, we demonstrated that resveratrol effectively attenuates HG-induced apoptosis of podocytes via the activation of autophagy in db/db mice. Importantly, our results suggested that the protective effects of resveratrol are mediated by the suppression of miR-383–5p. Thus, this study revealed a new possible strategy to protect podocytes against apoptosis in DN.

Resveratrol has a variety of biological activities; it has anti-inflammatory and antioxidant effects, it regulates lipid metabolism, and it improves microcirculation with antitumor effects. In the field of DN, resveratrol was shown to ameliorate renal injury and to enhance biogenesis of mitochondria with Mn-superoxide dismutase (Mn-SOD) dysfunction in the kidneys of db/db mice through improvement of oxidative stress via normalization of Mn-SOD function and glucose-lipid metabolism^23^. It is controversial whether resveratrol treatment can improve glycemic control and decrease insulin resistance. Some studies^24^,^25^ found resveratrol is able to improve glycemic control and insulin sensitivity. On the contrary, resveratrol did neither affect plasma glucose concentrations, nor decrease insulin resistance in other researches^26^,^27^. As well as our peivious work, in current study, resveratrol treatment did not affect blood glucose in db/db mice, suggesting that the beneficial effect of resveratrol on DN is independent of the blood glucose levels. As we all know, DN is often accompanied by high blood pressure and the control of blood pressure is closely related to the development of diabetic nephropathy. It is found that resveratrol could decrease blood pressure in many hypertension animal models, while, in our study, resveratrol did not affect changes in Systolic blood pressure (SBP) in db/db mice. Some other studies in models of type I and type II diabetic nephropathy (DN) also showed the same results and the mechanism needs to be further studied.

Although a body of evidence has shown that resveratrol protects against the development of DN, the underlying mechanism is not fully understood. To our knowledge, few studies have been conducted on the role of resveratrol in podocytes are available. Therefore, we focused this study on the role of resveratrol in podocytes. First, we found that resveratrol relieved a series of indicators of DN and attenuates podocyte apoptosis in db/db mice. Then, in human podocytes, we found HG induced podocyte apoptosis was in a dose- and time-dependent manner, while treatment with resveratrol strongly decreased expression of Cleaved caspase-3 and Bax in a dose-dependent manner. Flow-cytometric analysis also showed that the number of early apoptotic cells was increased by HG treatment, and down-regulated by resveratrol pretreatment. Together, these results illustrated that resveratrol protects podocytes in DN through the inhibition of apoptosis and improved DN. However, the exact mechanism remains to be unraveled by in-depth studies.

Recently, it has been reported that resveratrol (150–250 μmol/l) is sufficient to stimulate basal autophagic activity in cervical cancer cells, whereas in the absence of resveratrol, damaged organelles accumulated and energy homeostasis was disrupted, indicating that resveratrol plays important roles in the regulation of autophagy^28^. In the current study, we found that resveratrol ameliorated the abnormal autophagy level in db/db mice. In human podocytes, the level of autophagy was up-regulated in the initial stage, while it declined with resveratrol treatment time. LC3-II immunofluorescence staining, fluorescence microscopy of GFP-LC3 punctate structures, western blot analysis of autophagy-related proteins, and transmission electron microscopy indicated that autophagy was stimulated after exposure of podocytes to resveratrol and suppressed by bafilomycin A.

The crosstalk between autophagy and apoptosis is extremely complex. Autophagy is both protective against and a likely cause of cell death. Excessive and persistent autophagy will lead to loss of its protective effect and result in autophagic cell death. Increasing evidence shows that in diabetes, autophagic activity in the organs, especially in metabolic organs, decreases. The protective effect of autophagy in the kidneys has been shown through research on aging kidneys, and hypoxic and drug-induced renal injury in animal models^29^,^30^,^31^,^32^. In this study, to examine the role of resveratrol-induced autophagy in apoptosis of podocytes after HG exposure, autophagy was inhibited chemically with 3-MA and by using Atg5 shRNA. We found that 3-MA treatment led to reduced autophagy and increased apoptosis, in either the presence or absence of resveratrol. Similarly, Atg5 inhibition significantly increased apoptotic cells after resveratrol treatment. Thus, resveratrol attenuates HG-induced apoptosis in podocytes via the activation of autophagy.

The mechanism of regulation of autophagy by resveratrol at different stages in various organs and cell types is multifactorial. Park *et al*.^33^ demonstrated that resveratrol induces autophagy by directly inhibiting the mammalian target of rapamycin (mTOR)- Unc-51-like kinase-1 (ULK1) pathway. Other studies suggested that resveratrol attenuated tumor necrosis factor-α (TNF-α)-induced matrix metalloprotease-3 (MMP-3) expression in human nucleus pulposus cells by activating autophagy via the AMPK/SIRT1 signaling pathway^34^. In addition to intracellular signaling pathways, miRNAs have been shown to play a central role in autophagy regulation^18^,^19^. Tekirdag *et al*.^19^ reported miR181A (hsa-miR-181a-1) to be an autophagy-regulating miRNA; overexpression of miR181A resulted in the attenuation of starvation- and rapamycin-induced autophagy in MCFλ, Huh-7, and K562 cells. Moreover, antagomir-mediated inactivation of endogenous miRNA activity stimulated autophagy^19^. In our studies, using microarray analysis, we found that the level of miR-383-5p was significantly reduced in db/db mice treated with resveratrol as compared to db/db mice. Thus, we selected this miRNA for further study. Expression of miR-383-5p was suppressed after treatment with resveratrol in both db/db mice kidney and human podocytes. In addition, miR-383-5p overexpression inhibited LC3-I to LC3-II conversion. Further, overexpression of miR-383-5p significantly blocked the resveratrol-induced increase in autophagy and increased HG-induced apoptosis. Thus, we propose miR-383-5p as a novel autophagy-related microRNA. We will explore miR-383-5p target gene(s) and its exact mode of action in the regulation of autophagy in future studies.

Despite the important discoveries, there are also limitations to this study. In this study, we indicated that resveratrol effectively attenuates high glucose-induced apoptosis via the activation of autophagy in db/db mice and podocytes, which involves miR-383-5p, but we used the RT-PCR data from whole kidney homogenates to investigate the role of the miR-383-5p. The localisation of the expression of miR-383-5p in the kidney is not investigated, this needs to be further studied *in vivo* with a podocyte-specific method. Besides, the sample numbers were small, needed to be expanded. What’s more, the complex crosstalk between autophagy and apoptosis in DN was not investigated in depth; we will explore the possible molecular pathways of autophagy and apoptosis in future studies.

In conclusion, resveratrol was shown to have dramatic protective effects in podocytes of db/db mice and on cultured human podocytes through the reduction of apoptosis, and may be a potential drug for DN. Inhibition of autophagy by 3-MA and Atg5 shRNA reversed the protective effect of resveratrol on podocytes. Interestingly, our findings suggested miR-383-5p might play a role in the regulation of autophagy by resveratrol; this discovery may explain the prime mechanism of resveratrol. Further investigation of miR-383-5p target genes and signaling pathways is necessary to reveal the specific mechanism of resveratrol in modulating autophagy and protecting against DN.

## Materials and Methods

### Reagents and antibodies

Resveratrol, 3-MA, 4′,6-diamidino-2-phenylindole (DAPI), and paraformaldehyde were purchased from Sigma-Aldrich (St. Louis, MO, USA). BCA protein assay kit was obtained from Beyotime (Shanghai, China). RPMI-1640 medium, fetal bovine serum (FBS), insulin-transferrin-selenium, trypsin, penicillin, and streptomycin were obtained from Gibco (New York, NY, USA). Lipofectamine 2000 was obtained from Invitrogen Life Technologies (Grand Island, NY, USA). Antibodies against LC3-II and Beclin-1 were purchased from Cell Signaling (Beverly, MA, USA); antibodies against β-actin, cleaved caspase-3, and BAX were from Signalway Antibody (College Park, MD, USA); antibodies against Atg5 and p62 were from Abcam (Cambridge, UK); and antibody against nephrin was from Santa Cruz Biotechnology (Santa Cruz, CA, USA).Chemiluminescent HRP substrate was purchased from Millipore (Billerica, MA, USA). DyLight 594-labeled goat anti-rabbit IgG was purchased from Abbkine (Redlands, CA, USA). HRP-labeled goat anti-rabbit IgG was obtained from KeyGen Biotech (Nanjing, Jiangsu, China). Atg5 shRNA and negative control shRNA were purchased from GenePharma (Shanghai, China). Annexin-V FITC apoptosis detection kit was obtained from BD Biosciences (Franklin Lakes, NJ, USA). Microalbuminuria enzyme-linked immunosorbent assay (ELISA) kit was purchased from SenBejia Biotech (Nanjing, Jiangsu, China).

### Animal experiments

We used diabetic db/db and db/m mice with a C57BL/KsJ genetic background, which were obtained from the Mode Animal Centre of Nanjing University (Nanjing, China). Db/db mice were a genetic model of an early stage of type 2 diabetic nephropathy with hyperglycemia and urinary albumin excretion enhancement, while db/m mice were used as the control. The mice were housed in well-ventilated plastic cages with stainless steel grid tops at 22 ± 2 °C with a 12 h light/dark cycle. At 8 weeks of age, the mice were divided into three groups (db/m, db/db, and db/db + Res), each of which comprised 6 mice. The db/db + Res mice were given resveratrol by oral gavage at a dose of 10 mg/kg/day for 12 weeks. The db/m and db/db groups were given an equivalent amount of saline by oral gavage for the same period. The dosage was adjusted for body weight changes every week of the entire study period. Fasting blood glucose level (FBS) was measured every 2 weeks in all animals. Systolic blood pressure (SBP) was measured in conscious mice by tail cuff plethysmography using a BP-2000 blood pressure analysis system (Visitech Systems, Apex, NC). Blood pressures were measured 10 times per day for 4 consecutive days, and a mean value was generated for each individual mouse. Individual mice were placed in metabolic cages for 24-h urine collection. The urine samples were stored at −80 °C until analysis. At the end of the experimental period, blood samples were obtained from the retro-orbital plexus for biochemical assays, and the kidneys were harvested for histological assessments. All animal experiments were conducted in accordance with the committee guidelines of the Nanjing Medical University and approved by the IACUC (Institutional Animal Care and Use Committee of Nanjing Medical University, Ethical NO. 14030134).

### ELISA for 24-h microalbuminuria

Twenty-four-hour microalbuminuria was assessed using the ELISA kit according to the manufacturer’s instructions.

### Plasma biochemical analysis

Plasma samples were acquired by centrifugation of the blood samples at 1000 × *g,* at 4 °C and were stored at −80 °C until analysis. The levels of creatinine, and urea nitrogen were measured in the clinical laboratory of The Second Affiliated Hospital of Nanjing Medical University.

### Kidney histology and immunohistochemistry

The harvested kidneys were sent to the kidney laboratory of The Second Affiliated Hospital of Nanjing Medical University for HE and PAS staining. In addition, after deparaffinization, sections were placed in citrate-buffered solution (pH 6.0) and heated for antigen retrieval. Subsequently, the sections were incubated with anti-mouse primary antibody against nephrin overnight at 4 °C, followed by incubation with biotinylated secondary antibodies. Finally, diaminobenzidine tetrahydrochloride substrate was added to develop the reaction. Histologic evaluation was performed in a blinded manner using an Olympus microscopy system.

### Preparation of podocytes

Human podocytes^35^ were cultured in RPMI-1640 medium supplemented with 10% FBS, 1 × insulin-transferrin-selenium, 100 units/ml penicillin and 100 μg/ml streptomycin at a permissive temperature (33 °C), and they entered growth arrest after transfer to a non-permissive temperature (37 °C). After serum starvation for 16 h, the cells were exposed to the indicated conditions.

### Induction of autophagy/apoptosis in podocytes

To induce autophagy/apoptosis, cells were incubated with glucose at concentrations of 5.6–30 mmol/l (HG) for 0–48 h. Autophagy inhibitor 3-MA (3 mM) or bafilomycin A (10 nM) was added directly to the cultures before exposure to HG and resverstrol. The sequence of the Atg5 shRNA was GCTTCGAGATGTGTGGTTTGG, the sequence of the negative control shRNA was GTTCTCCGAACGTGTCACGT. Cells were transfected with Atg5 shRNA or the negative control shRNA using Lipofectamine 2000 according to the manufacturer’s instructions (Invitrogen). After transfection for 6 h, fresh RPMI-1640 medium containing 10% FBS was added. The cells were cultured for 24 h and incubated under control and experimental conditions for another 48 h. The shRNA efficiency was verified by western blotting.

### Autophagy measurement using GFP-LC3

Podocytes were plated in 24-well plates and incubated with fresh Opti-MEM reduced-serum medium 1 h before transfection. GFP-LC3 plasmid (Novobio, Shanghai, China) was transfected into the cells using Lipofectamine 2000. Transfected cells were cultured for 6 h, after which the medium was replaced with fresh RPMI-1640 containing 10% FBS. Twenty-four hours later, the GFP-LC3-transfected cells were incubated under control and experimental conditions for 48 h. The autophagy level was measured by counting of cells with GFP-LC3 punctate structures under a fluorescence microscope.

### Evaluation of autophagy by transmission electron microscopy

Mice kidney samples and human podocytes were washed and fixed in ice-cold glutaraldehyde (2.5% in 0.1 M phosphate buffer, pH 7.4) at 4 °C overnight, post-fixed in 1% osmium tetroxide for 3 h, dehydrated in an ascending gradual series of ethanol, infiltrated with propylene oxide, and embedded in epon resin. Samples were sliced into 70-nm ultrathin sections and stained with uranyl acetate. Representative areas were chosen for transmission electron microscopic analysis (JEOL-1010, Japan).

### Immunofluorescence assay

For immunofluorescence staining, 5-μm kidney cryosections were prepared and fixed in cold methanol/acetone (1:1) for 10 min. After being blocked with 2% normal goat serum in PBS for 40 min, the sections were incubated with primary antibodies against cleaved caspase-3 in PBS containing 1% BSA overnight at 4 °C. The sections were then washed thoroughly in PBS and incubated with DyLight 594-labeled goat anti-rabbit IgG (1:500) in the dark for 1 h at room temperature. After thorough washing with PBS, the slides were viewed under a fluorescence microscope equipped with a digital camera.

To detect and localize cleaved caspase-3 and LC3-II expression in cells, the cells were fixed in 4% paraformaldehyde and washed three times with PBS. Nonspecific sites were then blocked with normal goat serum for 2 h at room temperature. Next, the cells were incubated overnight with anti-cleaved caspase-3 and anti-LC3-II (1:500) at 4 °C. The cells were washed 3 times with PBS, followed by incubation with DyLight 594-labeled goat anti-rabbit IgG for 1 h at room temperature and washing 3 times with PBS. Finally, the cells were stained with DAPI to visualize the nuclei and analyzed using a fluorescence microscope. In each experimental setting, images were captured with identical light exposure parameters and aperture settings.

### Flow-cytometric analysis of cell apoptosis rate

Apoptosis of podocytes was quantified with an Annexin V-FITC apoptosis detection kit. Briefly, podocytes were trypsinized and resuspended in 1 × binding buffer and collected by centrifugation at 1000 × *g* for 5 min. The cell suspension was incubated with Annexin V-FITC and propidium iodide for 10–20 min at room temperature in the dark. Stained cells were immediately detected using a flow cytometer (FACSARIA II; BD Biosciences, San Jose, CA, USA).

### Western blot analysis

Human podocytes were harvested from culture dishes and lysed in RIPA buffer on ice for 30 min. The cell lysates were centrifuged at 12 000 × *g* for 15 min at 4 °C and the supernatants were used as total protein. The bicinchoninic acid (BCA) protein assay kit was used to determine the protein concentration. Equal amounts of protein sample (30 μg) were separated by SDS-PAGE and transferred to polyvinylidene fluoride (PVDF) membranes. The membranes were blocked with 5% non-fat milk in Tris Buffered Saline with Tween-20 (TBST) at room temperature for 2 h and then incubated overnight at 4 °C with the following primary antibodies: anti-β-actin, anti-p62, anti-LC3-II, anti-Atg5, anti-cleaved caspase-3, anti-Bax. HRP-labeled goat anti-rabbit IgG diluted at 1:20 000 in TBST was used as secondary antibody at room temperature for 1 h. β-actin was used as the protein loading control. Proteins bands were detected by chemiluminescent HRP substrate using a digital gel image analysis system (Tanon-4500; Tanon Science and Technology, Shanghai, China).

### Microarray analysis

Total RNA isolated from kidney samples of db/m, db/db, and db/db + Res mice was quantified with a NanoDrop ND-2100 (Thermo Scientific, Thermo Fisher Scientific, MA, USA) and RNA integrity was assessed using an Agilent 2100 bioanalyzer (Agilent Technologies). Sample labeling, microarray hybridization, and washing were performed according to the manufacturer’s standard protocols. Briefly, total RNA was tailed with poly-A and then labeled with biotin. The labeled RNAs were hybridized onto the Affymetrix miRNA 4.0 microarray. After washing and staining of the slides, the arrays were scanned with an Affymetrix Scanner 3000 (Affymetrix). Affymetrix GeneChip Command Console software (version 4.0, Affymetrix) was used to analyze array images to get raw data and for RMA normalization. GeneSpring software (version 12.5; Agilent Technologies) was used for data analysis. Differentially expressed miRNAs were identified on the basis of a fold-change ≥ 2.0.

### Real-time reverse transcriptase (qRT-)PCR for miR-383 quantification

Total RNA (mRNA and miRNA) of kidney samples and human podocytes was prepared using a TRIzol RNA isolation system (Invitrogen) according to the manufacturer’s instructions and reverse-transcribed to complementary DNA (cDNA) using a reverse transcription kit (Takara, Japan). Forward primers for hsa-miR-383 and mmu-miR-383 were purchased from GeneCopoeia (Guangzhou, Guangdong, China). The expression of miR-383 was measured by qRT-PCR with SYBR Green PCR Kit (Takara) on an Applied Biosystems StepOne-Plus real-time PCR system and human U6 RNA was amplified as an internal control. The relative expression ratio of miR-383 was calculated by the 2^−ΔΔCT^ method.

### Transfection of miR-383-5p mimics and inhibitors

The following were purchased from GenePharma (Shanghai, China): miR-383-5p mimics; Negative mimics, miR-383-5p inhibitors and Negative inhibitors. The sequence of the miR-383-5p mimics (5′ to 3′): sense AGAUCAGAAGGUGAUUGUGGCU antisense CCACAAUCACCUUCUGAUCUUU, the sequence of the Negative mimics (5′ to 3′): sense UUCUCCGAACGUGUCACGUTT antisense ACGUGACACGUUCGGAGAATT, the sequence of the miR-383-5p inhibitors (5′ to 3′): AGCCACAAUCACCUUCUGAUCU, the sequence of the Negative inhibitors (5′ to 3′): CAGUACUUUUGUGUAGUACAA. Transfection of miR-383-5p mimics and inhibitors, miR-383-5p mimics/inhibitors or negative mimics/inhibitors were transfected into the cells by using Lipofectamine 2000. After transfection for 6 h, fresh RPMI-1640 medium containing 10% FBS was added. The cells were cultured for 24 h and incubated under control and experimental conditions for another 48 h.

### Statistical analysis

All data are expressed as the mean ± SEM of at least three independent experiments. Differences between groups were analyzed using Student’s *t*-test and the level of significance was set at *P* < 0.05. The analyses were performed using SPSS 16.0 software.

## Additional Information

**How to cite this article:** Huang, S.-S. *et al*. Resveratrol protects podocytes against apoptosis via stimulation of autophagy in a mouse model of diabetic nephropathy. *Sci. Rep.* **7**, 45692; doi: 10.1038/srep45692 (2017).

**Publisher's note:** Springer Nature remains neutral with regard to jurisdictional claims in published maps and institutional affiliations.
